# Home capillary sampling and screening for type 1 diabetes, celiac disease, and autoimmune thyroid disease in a Swedish general pediatric population: the TRIAD study

**DOI:** 10.3389/fped.2024.1386513

**Published:** 2024-04-18

**Authors:** Maria Naredi Scherman, Alexander Lind, Samia Hamdan, Markus Lundgren, Johan Svensson, Flemming Pociot, Daniel Agardh

**Affiliations:** ^1^ Department of Clinical Sciences Malmö, Lund University, Malmö, Sweden; ^2^ Department of Pediatrics, Skåne University Hospital, Malmö, Sweden; ^3^ Department of Pediatrics, Kristianstad Central Hospital, Kristianstad, Sweden; ^4^ Department of Clinical Medicine, Faculty of Health and Medical Sciences, University of Copenhagen, Copenhagen, Denmark; ^5^ Department of Translational Type 1 Diabetes Research, Steno Diabetes Center Copenhagen, Herlev, Denmark

**Keywords:** autoimmune diseases, screening, type 1 diabetes, celiac disease, autoimmune thyroid disease, pediatrics

## Abstract

**Objective:**

To screen a general pediatric population for type 1 diabetes (T1D), celiac disease (CD), and autoimmune thyroid disease (AITD) after home capillary sampling.

**Methods:**

Swedish schoolchildren between 6–9 years and 13–16 years of age were invited to screening by taking a capillary sample at home. Samples were returned by mail and assessed for autoantibodies associated with T1D, CD, and AITD. Persistently autoantibody-positive children were referred for clinical follow-up.

**Results:**

Of 19,593 invited, 3,527 (18.0%) consented to participate and 2,315/3,527 (65.6%) returned a blood sample of sufficient volume. Hemolysis occurred in 830/2,301 (36.1%) samples. After exclusion of 42 children with previously known T1D, CD, or AITD, and two autoantibody-positive children who declined a confirmatory sample, 2,271/19,593 (11.6%) were included. 211/2,271 (9.3%) had persistent autoantibodies: 60/2,271 (2.6%) with T1D autoantibodies, 61/2,271 (2.7%) with CD autoantibodies, and 99/2,271 (4.4%) with AITD autoantibodies; 9/2,271 (0.4%) were autoantibody positive for ≥1 disease. After clinical follow-up, 3/2,271 (0.1%) were diagnosed with T1D, 26/2,271 (1.1%) with CD, and 6/2,271 (0.3%) with AITD. Children with a first-degree relative (FDR) with T1D, CD, and/or AITD, had higher occurrence of autoantibodies compared to children without an FDR (63/344, 18.3%, vs. 148/1,810, 8.2%) (*p* < 0.0001, OR 2.52, 95% CI 1.83–3.47), and higher occurrence of screening-detected diagnosis (14/344, 4.1%, vs. 21/1,810, 1.2%) (*p* < 0.0001, OR 3.61, 95% CI 1.82–7.18). Half of these children screened positive for another disease than the FDR.

**Conclusion:**

Screening for T1D, CD, and AITD by home capillary sampling in a Swedish general pediatric population detected autoimmunity in 9.3% and undiagnosed disease in 1.5%.

## 1 Introduction

In 1968, the World Health Organization (WHO) published Wilson & Jungner's ten principles for general population screening (1). These have since then been revised (2), and to date only a few rare conditions are being screened for in the pediatric population. In 2023, the Italian parliament passed a law on general population screening for type 1 diabetes (T1D) and celiac disease (CD) in children aged 1–17 years (3). Whether or not these autoimmune disorders qualify for screening is under debate and many questions remain on how such screening should be implemented.

A common phenomenon for many autoimmune diseases is the appearance of disease-related autoantibodies, detectable in the blood months or years prior to clinical onset (4). By autoantibody screening, early detection and treatment of clinical disease may prevent acute and long-term complications. Autoantibody screening can also detect individuals in pre-clinical stages, who could benefit from secondary prevention in clinical intervention studies with the goal to halt disease progression.

T1D, CD, and autoimmune thyroid disease (AITD) are three autoimmune diseases affecting children and adolescents of all ages. Two or three of the diseases can co-exist in the same individual, or in multiple family members (5, 6). The co-existence is partly explained by a shared genetic background of predisposing human leukocyte antigen (HLA) risk genotypes and non-HLA loci (7, 8). Several previous studies have screened for each disease separately (9–13), or for a combination of two of the diseases (14, 15), but no study has screened a general pediatric population for all three diseases simultaneously. By screening, diabetic ketoacidosis in children with T1D (16), nutrient deficiencies and poor growth in children with CD (17), and potentially irreversible effects on growth and development in children with AITD (18), could be prevented.

Although early detection and treatment are beneficial for the individual, the costs for the society and increased burden for the healthcare system to follow up autoantibody positive individuals should be carefully evaluated before implementing large-scale screening of the general population. Moreover, screening needs to be performed with caution and evaluated for feasibility and safety in order not to cause harm or excessive trauma, especially if it is performed in children. To alleviate the burden on the healthcare system, home capillary sampling may facilitate large-scale screening. In two previous screenings for T1D, the majority of participants responded that they would prefer home capillary sampling before venipuncture at healthcare centers (19, 20). Self-collected capillary samples could therefore be an alternative for future screening for autoimmune diseases in children.

The aim of the TRIAD study was to screen children from a general pediatric population for T1D, CD, and AITD and to evaluate the feasibility of home capillary sampling for future large-scale screenings.

## 2 Methods

### 2.1 Study design and participants

Between August 2021 and February 2022, 19,994 of 67,121 eligible children from two birth cohorts (6–9 years or 13–16 years) living in Skåne, the southernmost region of Sweden, were randomly selected from the Swedish Tax Agency and invited to participate in the study. The number of children invited from each municipality was proportional to population size. There were no exclusion criteria. Written information about the study, a consent form, and a questionnaire were sent by regular mail. The questionnaire contained two questions: (1) if the child had been diagnosed with T1D, CD, or AITD prior to screening, and (2) if the child had a first-degree relative (FDR) diagnosed with T1D, CD, or AITD. Families who consented to the study were sent a home capillary sampling kit, containing two Prolance™ Safety Lancets, together with written instructions and a link to an online instructional video. Between August 2021 and June 2022 blood samples were returned to the laboratory by regular mail. All samples of sufficient volume (250 μl) were analyzed for autoantibodies associated with T1D, CD, and AITD. Autoantibody-positive children were instructed to leave a confirmatory sample, either through home capillary sampling or as a venous sample at a local healthcare center. Persistently autoantibody-positive children, without previously known disease, were referred to a pediatrician for clinical follow-up. Symptoms were inquired for after information on autoantibody positivity had been given. Feasibility of home capillary sampling in children was assessed by the success rate of sample collection, the quality of collected blood samples, and reported adverse events. The TRIAD study was approved by the Swedish Ethics Review Appeals Board (Ö5—2021/3.1). All participants' legal guardians signed the written informed consent.

### 2.2 Autoantibody analyses

Radiobinding assays (RBA) were used to analyze insulin autoantibodies (IAA), glutamic acid decarboxylase autoantibodies (GADA), islet antigen 2 autoantibodies (IA2A), and zinc transporter 8 autoantibodies (ZnT8A) associated with T1D; tissue transglutaminase autoantibodies (tTGA) (both immunoglobulin A, IgA-tTG, and immunoglobulin G, IgG-tTG) associated with CD; and thyroid peroxidase autoantibodies (TPOA) associated with AITD (21, 22). Thyroglobulin autoantibodies (THGA), associated with AITD, were analyzed using paramagnetic particle chemiluminescent immunoassay (CLIA) according to the manufacturer's instructions (Shenzen Yhlo Biotech Co., Ltd.). Our laboratory participated in the Islet Autoantibody Standardization Program (IASP) 2023 workshop and the results are summarized in Supplementary Table S1.

### 2.3 Clinical follow-up

Clinical follow-up of persistently autoantibody-positive children began in December 2021 and ended in May 2023. In children with T1D autoantibodies, hemoglobin A1c (HbA1c) levels were measured and elevated levels together with an impaired oral glucose tolerance test (OGTT), were diagnostic for T1D (23). In children with tTGA, CD was diagnosed according to the guidelines of the Swedish Society of Gastroenterology for CD (24): if a biopsy showed Marsh score ≥2, or if serology showed a level of IgA-tTG >10 times the upper level of normal in two consecutive samples. The Swedish guidelines are based on the European Society for Pediatric Gastroenterology, Hepatology, and Nutrition (ESPGHAN) guidelines for CD (25), with the exception that endomysial antibodies are not required for diagnosis (24). In children with AITD autoantibodies, thyroid stimulating hormone (TSH) was measured, and a diagnosis of AITD was established if TSH levels were deranged in combination with an ultrasonography with signs of thyroiditis (26). Caregivers to children with persistent autoantibody positivity, but no signs of clinical disease, received oral and written information on the child's risk of T1D, CD, and/or AITD, and which symptoms should prompt medical care. All autoantibody-positive children will have a clinical follow-up after one year, and children with multiple T1D autoantibodies will have clinical follow-up every three months.

### 2.4 Statistical analysis

Children with positive autoantibodies in both the screening and the confirmatory sample were classified as autoantibody positive. Children negative in screening, or positive in the screening but negative in the confirmatory sample, were classified as autoantibody negative. The primary outcome was autoantibody positivity for autoantibodies associated with T1D, CD, or AITD. The secondary outcome was screening-detected diagnosis of T1D, CD, or AITD. Statistical analyses were performed using IBM® SPSS® Statistics, version 29. The one-sample proportion test was used to test inequalities in the distribution of sex and age groups. The chi-square test, or Fisher's exact test for sample sizes of <5, were used to test whether there was a difference in prevalence between groups. Statistical significance was set at *p* < 0.05.

## 3 Results

### 3.1 Recruitment of study participants

**Figure 1:**
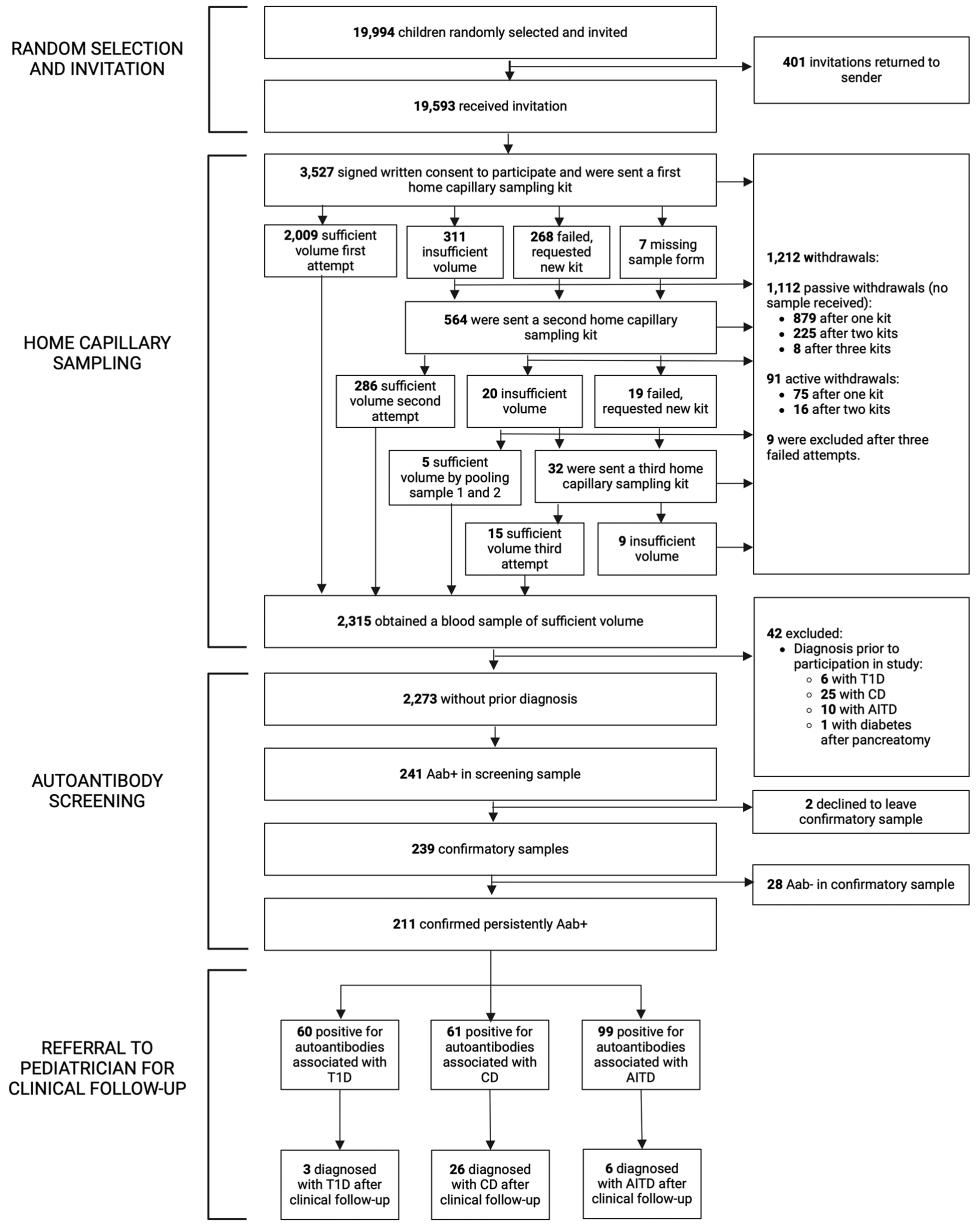
Flowchart of the TRIAD-study. Aab+, autoantibody positive; Aab-, autoantibody negative; T1D, type 1 diabetes; CD, celiac disease; AITD, autoimmune thyroid disease.

Of 67,121 eligible children, 19,994/67,121 (29.8%) were randomly selected and 19,593/67,121 (29.2%) received invitation and information about the study. In total, 3,527/19,593 (18.0%) consented to participate and were sent a home capillary sampling kit. A blood sample of sufficient volume was provided from 2,315/19,593 (11.8%) of invited participants, or 2,315/3,527 (65.6%) of those who gave consent to participate in the screening. Forty-one of 2,315 children (1.8%) reported having one of the diseases prior to screening; 6/2,315 (0.3%) had T1D, 25/2,315 (1.1%) had CD, and 10/2,315 (0.4%) had AITD. An additional child had diabetes after pancreatectomy. The samples from these 42 children were analyzed and results were reported to the families, but they were excluded from further statistical analysis. After excluding children with prior diagnosis, 2,273/19,593 (11.6%) invited children were included (Figure 1). Baseline characteristics are summarized in Supplementary Table S2.

### 3.2 Home capillary sampling

All children that consented to participation received a home capillary sampling kit and 2,009/3,527 (57.0%) succeeded to provide a blood sample of sufficient volume on the first attempt. Due to insufficient volume, failed attempts, or missing sample forms, 564/3,527 (16.0%) children received a second kit, and 32/3,527 (0.9%) received a third kit (Figure 1). No more than three attempts to obtain a blood sample were given. Of the 1,212/3,527 (34.4%) children that did not return a blood sample, 9/3,527 (0.3%) were excluded after three failed attempts, 91/3,527 (2.6%) actively withdrew from the study (the main reason being concerns about blood draw, reported in 56/91, 61.5%, of the children), and 1,112/3,527 (31.5%) samples were unreturned without stating why (passive withdrawals). No attempts to contact these families were made. Six of 3,527 (0.2%) children reported fainting during home capillary sampling. Time between sample draw date and process date was registered in 2,236 samples and was median 2 days (range 1–12 days, IQR 2–3 days). Presence of hemolysis was reported for 2,301 samples, of which 1,471/2,301 (63.9%) had no hemolysis and 830/2,301 (36.1%) had signs of hemolysis; 620/2,301 (26.9%) had slight or moderate hemolysis, and 210/2,301 (9.1%) had gross hemolysis (Supplementary Figure S1). Samples with hemolysis had a higher prevalence of IAA (24/830, 2.9%) compared to samples without hemolysis (22/1,471, 1.5%) (*p* = 0.022, OR 1.96, 95% CI 1.09–3.52) and a lower prevalence of tTGA (16/830, 1.9%) compared to samples without hemolysis (51/1,471, 3.5%) (*p* = 0.035, OR 0.55, 95% CI 0.31–0.97). Autoantibody prevalence in samples with and without hemolysis is summarized in Supplementary Table S3.

### 3.3 Outcome of screening

#### 3.3.1 Overall outcome of screening

**Figure 2:**
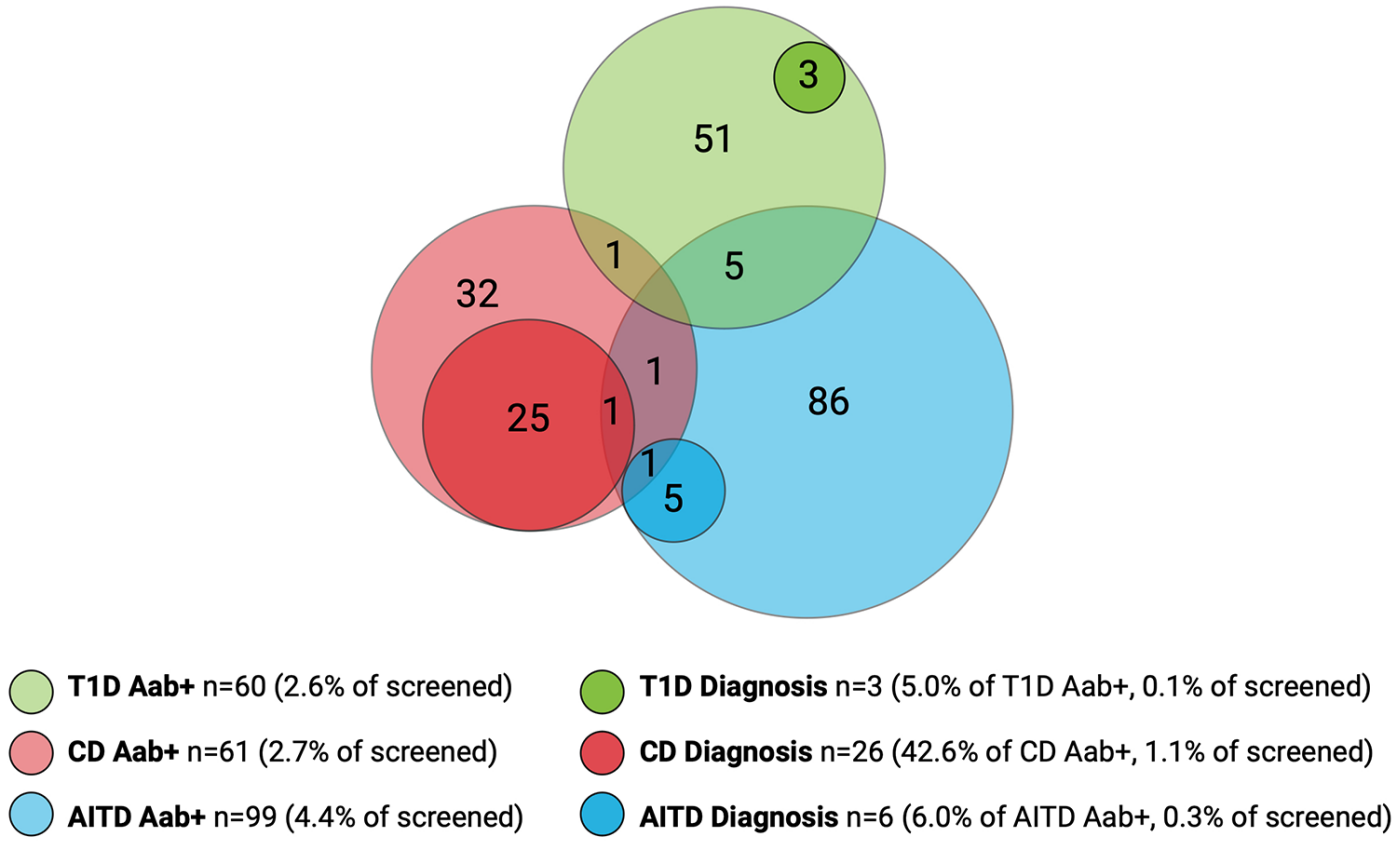
Overlap of autoantibody-positive children (Aab+) and children with screening-detected disease. Number of screened children, *n* = 2,271. Number of Aab+ children, *n* = 211. Number of children with screening-detected diagnosis, *n* = 35. T1D, type 1 diabetes; CD, celiac disease; AITD, autoimmune thyroid disease.

Among the children included in screening, 241/2,273 (10.6%) were positive for at least one autoantibody and were instructed to leave a confirmatory sample. Confirmatory samples were received at median 113 days after screening (range 56–449 days, IQR 94–135 days). Two of the tTGA-positive children declined to leave a confirmatory sample and were excluded as they could not be classified as persistently positive or negative. Thus, a total of 2,271 children were included in further statistical analysis. Of the confirmatory samples, 211/239 (88.3%) were autoantibody positive, yielding a persistent autoantibody positivity rate of 211/2,271 (9.3%) (Figure 1). Nine of 2,271 (0.4%) children were autoantibody positive for >1 disease (one child for T1D and CD, five children for T1D and AITD, three children for CD and AITD) (Figure 2). Autoantibody positivity was higher among females (134/1,191, 11.3%) than among males (77/1,080, 7.1%) (*p* = 0.0007, OR 1.65, 95% CI 1.23–2.21) (Table 1). After clinical follow-up, 35/211 (16.6%) autoantibody-positive children were diagnosed with T1D, CD, or AITD, resulting in a 35/2,271 (1.5%) rate of screening-detected disease (Figure 1).

**Table 1:** Number of autoantibody-positive children in different subgroups.

	No. (%)	Aab+	*p*-value	T1D	*p*-value	CD	*p*-value	AITD	*p*-value
			OR	Aab+	OR	Aab+	OR	Aab+	OR
		No. (%)	(95% CI)	No. (%)	(95% CI)	No. (%)	(95% CI)	No. (%)	(95% CI)
All screened	*n *= 2,271	211 (9.3)		60 (2.6)		61 (2.7)		99 (4.4)	
Sex	*n *= 2,271								
Female	1,191 (52.4)	134 (11.3)	*p *= 0.0007* OR 1.65 (1.23–2.21)	33 (2.8)	*p *= 0.69 OR 1.11 (0.66–1.86)	37 (3.1)	*p *= 0.19 OR 1.41 (0.84–2.37)	72 (6.0)	*p* < 0.0001* OR 2.51 (1.60–3.94)
Male	1,080 (47.6)	77 (7.1)		27 (2.5)		24 (2.2)		27 (2.5)	
Age group	*n *= 2,271								
6–9 years	1,087 (47.9)	109 (10.0)	*p *= 0.25 OR 1.18 (0.89–1.57)	38 (3.5)	*p *= 0.015* OR 1.91 (1.12–3.26)	45 (4.1)	*p* < 0.0001* OR 3.15 (1.77–5.61)	29 (2.7)	*p *= 0.0002* OR 0.44 (0.28–0.68)
13–16 years	1,184 (52.1)	102 (8.6)		22 (1.9)		16 (1.4)		70 (5.9)	
6–9 years	*n *= 1,087								
Female	549 (50.5)	70 (12.8)	*p *= 0.0025* OR 1.87 (1.24–2.82)	20 (3.6)	*p *= 0.79 OR 1.09 (0.57–2.09)	30 (5.5)	*p *= 0.027 OR 2.02 (1.07–3.79)	23 (4.2)	*p *= 0.0017 OR 3.88 (1.57–9.60)
Male	538 (49.5)	39 (7.2)		18 (3.3)		15 (2.8)		6 (1.1)	
13–16 years	*n *= 1,184								
Female	642 (54.2)	64 (10.0)	*p *= 0.071 OR 1.47 (0.97–2.23)	13 (2.0)	*p *= 0.64 OR 1.22 (0.52–2.89)	7 (1.1)	*p *= 0.40 OR 0.65 (0.24–1.77)	49 (7.6)	*p *= 0.0063 OR 2.05 (1.21–3.46)
Male	542 (45.8)	38 (7.0)		9 (1.7)		9 (1.7)		21 (3.9)	
FDR with T1D, CD, and/or AITD	*n *= 2,154								
Yes	344 (16.0)	63 (18.3)	*p* < 0.0001 OR 2.52 (1.83–3.47)	18 (5.2)	*p *= 0.0026 OR 2.32 (1.32–4.09)	22 (6.4)	*p* < 0.0001 OR 3.10 (1.82–5.30)	28 (8.1)	*p *= 0.0006 OR 2.17 (1.38–3.42)
No	1,810 (84.0)	148 (8.2)		42 (2.3)		39 (2.2)		71 (3.9)	

Aab+, autoantibody positive; T1D, type 1 diabetes; CD, celiac disease; AITD, autoimmune thyroid disease; FDR, first-degree relative.

* *p* < 0.05.

#### 3.3.2 Occurrence of T1D autoimmunity and T1D

**Figure 3:**
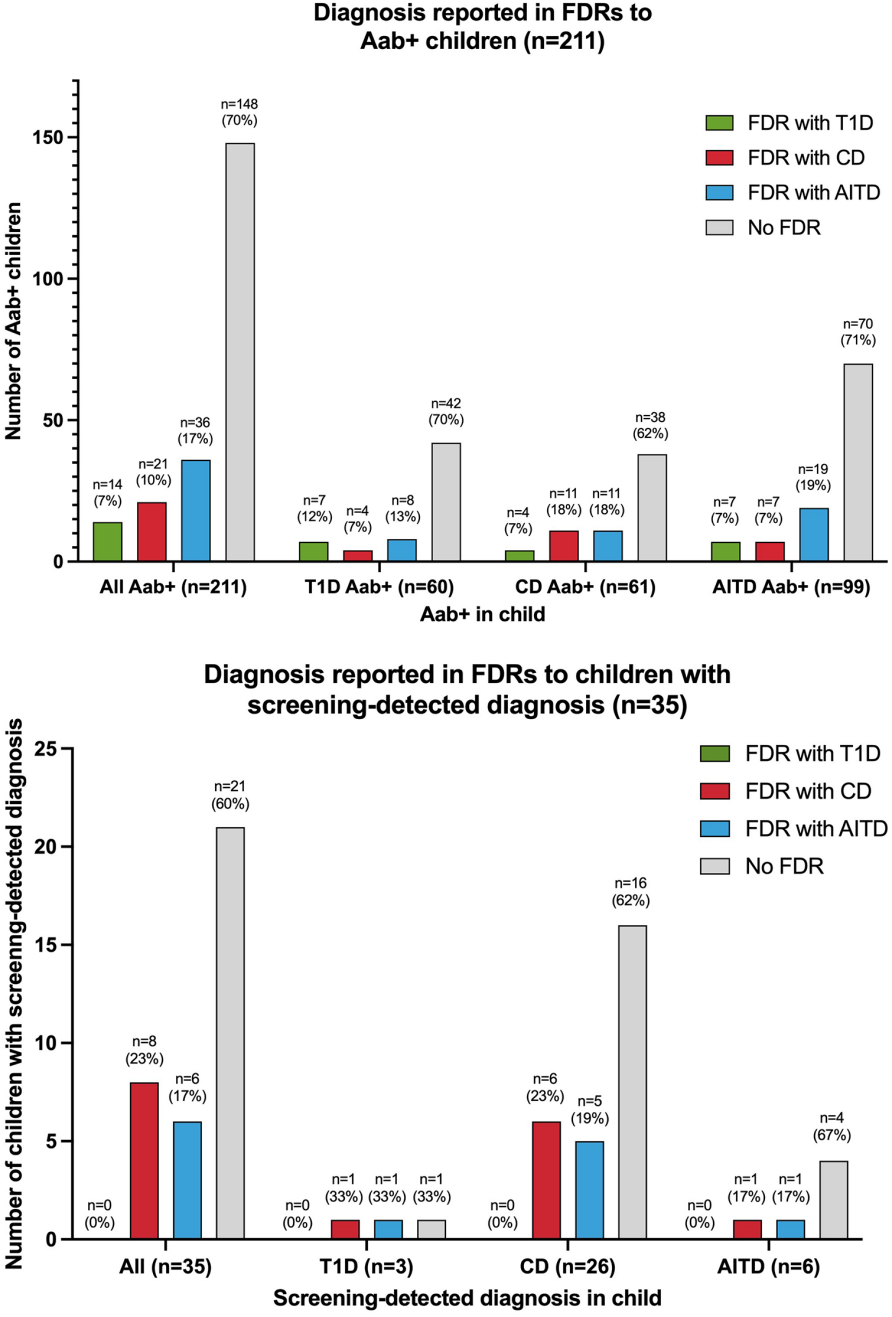
Diagnosis in first-degree relatives (FDRs) of autoantibody-positive (Aab+) children and children with screening-detected diagnosis T1D, type 1 diabetes; CD, celiac disease; AITD, autoimmune thyroid disease.

Sixty of the 2,271 (2.6%) screened children were persistently T1D autoantibody positive (Figure 2). The distribution of T1D autoantibodies is presented in Supplementary Table S4. Forty-six of 2,271 (2.0%) were positive for one T1D autoantibody and 14/2,271 (0.6%) were positive for two or more T1D autoantibodies. The occurrence of T1D autoimmunity was higher in the 6-to 9-year-old group (38/1,087, 3.5%) than in the 13-to 16-year-old group (22/1,184, 1.9%) (*p* = 0.0015, OR 1.91, 95% CI 1.12–3.26) (Table 1). Three of the 60 T1D autoantibody-positive children (5.0%) had elevated HbA1c levels (>42 mmol/mol) and an impaired OGTT and were diagnosed with T1D (Figure 2). These three children were all in the 6-to 9-year-old group and persistently positive for all four T1D autoantibodies. None of these children had clinical symptoms of T1D or diabetic ketoacidosis; none had an FDR with T1D, but one had an FDR with CD, and one had an FDR with AITD (Figure 3). Screening-detected T1D was estimated at 0.1% (3/2,271).

#### 3.3.3 Occurrence of CD autoimmunity and CD

Sixty-one of the 2,271 (2.7%) screened children were persistently positive for either IgA-tTG or IgG-tTG (Figure 2). The distribution of IgA-tTG and IgG-tTG are presented in Supplementary Table S4. Sixteen of 61 (26.2%) were positive only for either IgA-tTG (8/61, 13.1%) or IgG-tTG (8/61, 13.1%), and 45/61 (73.8%) were positive for both. Three of 61 (4.9%) tTGA-positive children had IgA deficiency. The occurrence of CD autoimmunity was higher in the 6- to 9-year old group (45/1,087, 4.1%) compared to the 13- to 16-year-old group (16/1,184, 1.4%) (*p* < 0.0001, OR 3.15, 95% CI 1.77–5.61), and in the 6- to 9-year-old group the occurrence was higher among females (30/549, 5.5%) than among males (15/538, 2.8%) (*p* = 0.027, OR 2.02, 95% CI 1.07–3.79) (Table 1). Twenty-six of 61 (42.6%) tTGA-positive children were diagnosed with CD (Figure 2), of whom 10/26 (38.5%) were diagnosed after intestinal biopsy and 16/26 (61.5%) were serologically diagnosed; 18/26 (69.2%) were in the younger age group; 10/26 (38.5%) were asymptomatic at diagnosis; 6/26 (23.1%) had an FDR with CD and 5/26 (19.2%) had an FDR with AITD (Figure 3). Screening-detected CD was estimated at 1.1% (26/2,271).

#### 3.3.4 Occurrence of AITD autoimmunity and AITD

Ninety-nine of the 2,271 (4.4%) screened children were positive for AITD autoantibodies (Figure 2). The distribution of TPOA and THGA are shown in Supplementary Table S4. Fifty-eight of 99 (58.6%) were positive for only one AITD autoantibody; 13/99 (13.1%) were positive only for TPOA, 45/99 (45.5%) only for THGA, and 41/99 were positive for both (41.4%). The occurrence of AITD autoimmunity was higher among females (72/1,191, 6.0%) than males (27/1,080, 2.5%) (*p* < 0.0001, OR 2.51, 95% CI 1.60–3.94), and lower in the 6-to 9-year-old group (29/1,087, 2.7%) than in 13-to 16-year-old group (70/1,184, 5.9%) (*p* = 0.0002, OR 0.44, 95% CI 0.28–0.68) (Table 1). Six of the 99 (6.1%) children with AITD autoantibodies had elevated TSH levels and signs of thyroiditis on ultrasonography and were diagnosed with AITD (Figure 2). Three of the six children with AITD were from the younger age group (50%), and three from the older age group (50%). None had clinical symptoms of AITD. One of the six children (16.7%) had an FDR with AITD, and one of the six (16.7%) had an FDR with CD (Figure 3). Screening-detected AITD was estimated at 0.3% (6/2,271).

### 3.4 FDRs with T1D, CD, and/or AITD

Among the screened children, 2,154/2,271 (94.8%) answered the questionnaire and 344/2,154 (16.0%) reported having an FDR with T1D, CD, and/or AITD. The occurrence of autoantibodies was higher in children who reported an FDR with diagnosis (63/344, 18.3%), than in children that did not have an FDR with diagnosis (148/1,810, 8.2%) (*p* < 0.0001, OR 2.52, 95% CI 1.83–3.47). The occurrence of screening-detected diagnosis was also higher in children with an FDR with diagnosis (14/344, 4.1%), compared with children without any of the diseases in the family (21/1,810, 1.2%) (*p* < 0.0001, OR 3.61, 95% CI 1.82–7.18) (Table 1). Of the autoantibody-positive children with an FDR with diagnosis, 35/63 (55.6%) screened positive for autoantibodies associated with the same disease as reported in their FDR, 33/63 (52.4%) screened positive for autoantibodies associated with a different disease than the disease reported in the FDR; five of these 63 children (7.9%) screened positive for autoantibodies associated with both the same and a different disease than reported in the FDR. Seven of 14 children (50%) with screening-detected diagnosis were diagnosed with the same disease as their FDR; six of them with CD and one with AITD. The distribution of autoantibody-positive children and children with screening-detected diagnosis by FDR diagnosis is shown in Figure 3. In the 63 children with positive autoantibodies and an FDR with diagnosis, AITD was the most common diagnosis among the 67 FDRs (36/67 FDRs, 53.7%), AITD was also the most common diagnosis among mothers (33/47 mothers with diagnosis, 70.2%); T1D was the most common diagnosis in fathers (6/12 fathers with diagnosis, 50%); and CD was the most common diagnosis in siblings (5/8 siblings with diagnosis, 62.5%).

## 4 Discussion

In recent years, several initiatives for screening the general pediatric population for T1D and CD have emerged (3, 13, 15). However, before considering screening for these autoimmune diseases it is important to determine whether the conventionally accepted and established WHO principles for screening of the general population are fulfilled (2).

Firstly, the disease should be an important health problem with a high incidence or prevalence, and there should be a detectable latent or pre-clinical phase (2). The present study screened Swedish children randomly selected from a general population for three of the most common autoimmune diseases in childhood, T1D, CD, and AITD, and found that 9.3% had autoantibodies associated with any of the three diseases; 2.6% associated with T1D, 2.7% with CD, and 4.4% with AITD. The occurrence of multiple T1D autoantibodies was 0.6%. Autoantibodies were more frequently found in females (11.3%) and in children with an FDR with any of the three diseases (18.3%). After clinical follow-up, 1.5% of the screened children were diagnosed with one of the diseases: 1.1% with CD, 0.3% with AITD, and 0.1% with T1D. In the original cohort of 2,315 children providing a capillary sample, 1.1% had already been diagnosed with CD, 0.4% with AITD, and 0.3% with T1D, prior to screening. Thus, for every child with known CD or AITD in this cohort, an equal number of children were diagnosed through screening, and for every three children diagnosed with clinical T1D, an additional child with T1D was detected.

Previous screenings conducted in general pediatric populations reported a lower prevalence of T1D autoantibodies (around 2%) (9, 14), and multiple T1D autoantibodies (0.2%–0.5%) (9, 13, 14). The prevalence of CD autoantibodies (tTGA) in previous screening studies has been lower (0.8%–1.2%) (10, 27), similar (2.4%) (15), or higher (3.5%) (14) compared with our results. The prevalence of TPOA of 2.4% and THGA of 3.8% in the present study was comparable to the prevalence found in Finnish and Spanish school children (2.3%–2.6% for TPOA and 3.0%–3.4% for THGA) (11, 12). Discrepancies in results between the present study and previous studies are likely caused by two explanations. The higher occurrence of autoantibodies associated with T1D and CD found in our study could be explained by the fact that Sweden has the second highest incidence of T1D worldwide (28), and that CD prevalence in Sweden has been reported to reach almost 3% (29), compared with the global prevalence of CD in children of 0.9% (30). The fact that 16.0% of screened children had an FDR with T1D, CD, and/or AITD may have resulted in an overestimation of children with positive autoantibodies. Even so, the observation that autoantibodies were found in 8.2%, and screening-detected disease in 1.2%, of children without an FDR indicate that there is still a high frequency of autoimmunity and unrecognized disease in the Swedish general pediatric population. As expected, the occurrence of autoantibodies and screening-detected diagnosis was significantly higher in children with an FDR with any of the diseases, but interestingly, half of the autoantibody-positive children and children with screening-detected diagnosis screened positive for another disease than the diagnosis reported in their FDR. This supports the notion that family members of patients with T1D, CD, or AITD may benefit from screening for all three diseases. However, if the present screening would have only invited FDRs to patients with T1D, CD, or AITD for autoantibody testing, 60%–70% of the detected children would have been missed. Moreover, if the children would only have been screened for the same disease as the relative, approximately 80% of the children at risk or with unrecognized diagnosis would have been missed.

When screening the general population, the target population should be clearly defined, for example by an appropriate target age range (2). The peak incidence for autoantibodies associated with T1D is around 1–2 years of age for IAA and 3–5 years of age for GADA (31). The peak incidence for tTGA is at 2–3 years of age (32). It is possible that the occurrence of T1D and CD autoantibodies would have been even higher if screening was performed on preschool children instead of young school children. Longitudinal studies of the natural history of T1D and CD autoimmunity have demonstrated that a significant proportion of healthy children have autoantibodies which may wax and wane over a period of time before the onset of clinical disease (33, 34). This phenomenon of fluctuating or transient autoantibodies seems to be more prevalent during early childhood and declines with age. This may explain why we in the present screening found more children with autoantibodies associated with T1D and CD in the younger children aged 6–9 years compared with the older children aged 13–16 years. Conversely, we found AITD autoantibodies more frequently in children aged 13–16 years. This was expected as the risk of AITD increases with age (35). However, our results show that AITD autoantibodies may develop long before puberty, and half of the children diagnosed with AITD in our study were 6–9 years old.

When conducting screening in the general population, there should be an appropriate test acceptable to the target population (2). The feasibility and tolerability of autoantibody screening by self-collected capillary samples has been evaluated in a few previous studies (19, 20, 36). In one study 82% preferred home capillary sampling over venipuncture in an outpatient setting, and the percentage was even higher (90%) for children <8 years (36). Although home capillary sampling could reduce the costs of testing, as opposed to samples collected by healthcare professionals at research- or healthcare-centers, it cannot be at the expense of quality. In the present study, two thirds (65.6%) of the children who received a home capillary sampling kit managed to provide a blood sample of sufficient volume. In 9.1% of these samples there was gross hemolysis with risk of false positive results for IAA (37). However, the risk of a positive result due to hemolysis was reduced by requesting a confirmatory sample in all autoantibody-positive children. In the present study, the median time from sample draw date to process date was only two days, indicating that a delay in shipment or delivery was probably not the main cause of hemolysis. A plausible cause for hemolysis is an incorrect sampling technique, for instance by squeezing the finger to receive sufficient blood volume. For the present screening at least 250 μl blood was required for the analysis of seven autoantibodies using single-plex radiobinding assays and one autoantibody using CLIA. We speculate that the large volume needed can explain both the difficulty of reaching sufficient volume as well as the large number of samples with hemolysis. Indeed, using automated multiplex assays with the ability to screen large number of samples for multiple autoantibodies in a small volume of blood will most likely facilitate for effective screenings. Home capillary sampling was not associated with any severe adverse events, but the potential individual, societal and economic benefits of self-collected samples warrant careful evaluation in future studies.

There are some study weaknesses that limit the conclusions of the present screening. No information was available on why the majority of invited children and families did not consent to participation, or why one third of consenting families did not return a blood sample. The low participation rate of 11.6%, and the high participation rate of children with an FDR with T1D, CD, and/or AITD, thus limits conclusions to be drawn on the true prevalence of autoimmunity for the three diseases in the general pediatric population. Previous general population screenings recruited children attending regular well-baby visits (13), general pediatric practices (14), or local health fairs (9), resulting in participation rates of 22%, 26% and 90%, respectively. In these studies, oral and personal information about screening from pediatricians or research staff, as opposed to written information in our study, might have contributed to a higher participation rate.

In conclusion, autoantibodies associated with T1D, CD, and AITD were detected in 9.3% of Swedish schoolchildren, and 1.5% were subsequently diagnosed with one of the diseases. Both autoimmunity and screening-detected diagnosis were more common in children with an FDR with T1D, CD, or AITD, but in half of these children the autoantibody positivity or diagnosis detected in the child differed from the diagnosis reported in the FDR, confirming the overlap of the three diseases within families. However, limiting screening to FDRs will miss most children with increased risk of disease. Home capillary sampling has the potential to be used for large-scale screenings, but the diagnostic accuracy of new multiplex assays requiring a very small volume of blood, and the safety and tolerability of self-collected samples, must be further studied before it can be implemented in large-scale population-based screenings.

## Data Availability

The datasets presented in this article are not readily available because for the integrity of study participants, data collected for the study will not be officially available to others. Requests to access the datasets should be directed to maria.naredi_scherman@med.lu.se.
