# Supplementary material for: Home capillary sampling and screening for type 1 diabetes, celiac disease, and autoimmune thyroid disease in a Swedish general pediatric population: the TRIAD study
Source: Front Pediatr. 2024 Apr 18;12:1386513. doi: 10.3389/fped.2024.1386513 (PMC11063237; doi:10.3389/fped.2024.1386513)
Supplement: Supplementary file 1 [file Datasheet1.pdf]

Supplementary Material

Home Capillary Sampling and Screening for Type 1 Diabetes, Celiac Disease, and Autoimmune Thyroid Disease in a Swedish General Pediatric Population: The TRIAD Study

Maria Naredi Scherman*, Alexander Lind, Samia Hamdan, Markus Lundgren, Johan Svensson, Prof. Flemming Pociot, Prof. Daniel Agardh

*** Correspondence:** Maria Naredi Scherman, maria.naredi_scherman@med.lu.se

1 Supplementary Figures and Tables

1.1 Supplementary Figures

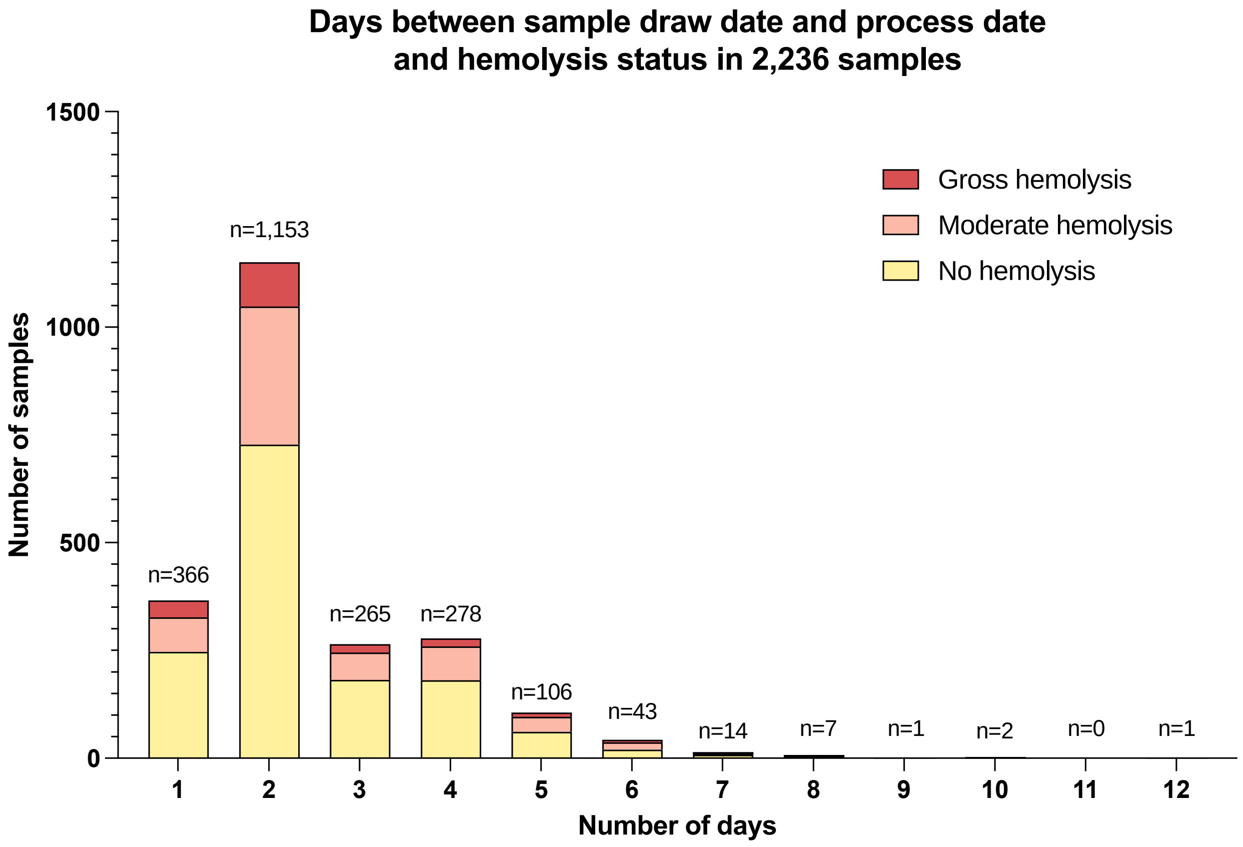

Supplementary Figure 1. Time between draw date and arrival date (process date) in laboratory, and quality of samples received after home capillary sampling. Draw date was reported in 2,236 samples of sufficient volume. Median 2 days (range 1-12 days, IQR 2-3 days).

1.2 Supplementary Tables

Supplementary Table 1. Islet Autoantibody Standardization Program (IASP) Workshop 2023.

Our laboratory's results from IASP Workshop 2023. Glutamic acid decarboxylase autoantibodies (GADA), islet-antigen-2 autoantibodies (IA-2A), insulin autoantibodies (IAA), zinc transporter 8 autoantibodies (ZnT8A).

**Autoantibody**	**Method**	**% Sensitivity**	**% Specificity**
GADA	Radiobinding Assay (RBA) Local	74.0	97.8
IA2A	Radiobinding Assay (RBA) Local	76.0	100.0
IAA	Radiobinding Assay (RBA) Local	28.0	98.9
ZnT8A	CR-RBA (C-Terminal arginine)	54.0	100.0
ZnT8A	CW-RBA (C-Terminal tryptophan)	56.0	100.0
ZnT8A	CQ-RBA (C-Terminal glutamine)	44.0	100.0

Supplementary Table 2. Baseline characteristics of invited, consenting, and screened children.

Differences in sex, age group and first-degree relative (FDRs) with type 1 diabetes (T1D), celiac disease (CD) and/or autoimmune thyroid disease (AITD). Since only families consenting to participation filled in the questionnaire, results of reported diagnosis in FDRs can only be presented for the consenting and screened. *p<0.05.

	**Invited No. (%)**	*p-value*	**Consenting No. (%)**	*p-value*	**Screened No. (%)**	*p-value*
**Sex**	**n=19,593**		**n=3,527**		**n=2,271**	
Female	9,574 (48.9)	p=0.0015*	1,801 (51.1)	p=0.21	1,191 (52.4)	p=0.021*
Male	10,019 (51.1)		1,726 (48.9)		1,080 (47.6)	
**Age group**	**n=19,593**		**n=3,527**		**n=2,271**	
6-9 years	10,038 (51.2)	p=0.0006*	1,791 (50.8)	p=0.36	1,087 (47.9)	p=0.044*
13-16 years	9,555 (48.8)		1,736 (49.2)		1,184 (52.1)	
**FDR with T1D, CD, and/or AITD**	-	-	**n=3,174**		**n=2,154**	
Yes	-	-	529 (16.7)	p<0.0001*	344 (16.0)	p<0.0001*
No	-	-	2,645 (83.3)		1,810 (84.0)	

Supplementary Table 3. Autoantibodies in capillary screening samples with and without hemolysis.

Type 1 diabetes (T1D), autoantibody-positive (Aab+), insulin autoantibodies (IAA), glutamic acid decarboxylase autoantibodies (GADA), islet-antigen-2 autoantibodies (IA-2A), zinc transporter 8 autoantibodies (ZnT8A), celiac disease (CD), immunoglobulin A-tissue transglutaminase (IgA-tTG), immunoglobulin G-tissue transglutaminase (IgG-tTG), autoimmune thyroid disease (AITD), thyroid peroxidase autoantibodies (TPOA), thyroglobulin autoantibodies (THGA). *p<0.05.

	**All (n=2,301)**	**No hemolysis (n=1,471)**	**Hemolysis (n=830)**	**p-value**	**OR**	**95% CI**
**Screening T1D Aab+**	**83 (3.6%)**	**44 (3.0%)**	**39 (4.7%)**	**p=0.035***	**1.60**	**1.03-2.48**
IAA	46 (2.0%)	22 (1.5%)	24 (2.9%)	p=0.022*	1.96	1.09-3.52
GADA	47 (2.0%)	29 (2.0%)	18 (2.2%)	p=0.75	1.10	0.61-2.00
IA-2A	11 (0.5%)	9 (0.6%)	2 (0.2%)	p=0.35	0.39	0.09-1.82
ZnT8A	10 (0.4%)	8 (0.5%)	2 (0.2%)	p=0.35	0.44	0.09-2.09
**Screening CD Aab+**	**67 (2.9%)**	**51 (3.5%)**	**16 (1.9%)**	**p=0.035***	**0.55**	**0.31-0.97**
IgA-tTG	60 (2.6%)	44 (3.0%)	16 (1.9%)	p=0.12	0.64	0.36-1.14
IgG-tTG	57 (2.5%)	43 (2.9%)	14 (1.7%)	p=0.067	0.57	0.31-1.05
**Screening AITD Aab+**	**119 (5.2%)**	**73 (5.0%)**	**46 (5.5%)**	**p=0.55**	**1.12**	**0.77-1.64**
TPOA	62 (2.7%)	37 (2.5%)	25 (3.0%)	p=0.48	1.20	0.72-2.01
THGA	101 (4.4%)	64 (4.4%)	37 (4.5%)	p=0.90	1.03	0.68-1.55

Supplementary Table 4 Autoantibodies in 2,271 screened children.

Autoantibody-positive (Aab+), type 1 diabetes (T1D), insulin autoantibodies (IAA), glutamic acid decarboxylase autoantibodies (GADA), islet-antigen-2 autoantibodies (IA-2A), zinc transporter 8 autoantibodies (ZnT8A), celiac disease (CD), immunoglobulin A-tissue transglutaminase (IgA-tTG), immunoglobulin G-tissue transglutaminase (IgG-tTG), autoimmune thyroid disease (AITD), thyroid peroxidase autoantibodies (TPOA), thyroglobulin autoantibodies (THGA).

	**No. (%)**
**All screened**	**2,271 (100)**
**Aab+**	**211 (9.3)**
**T1D Aab+**	**60 (2.6)**
IAA	30 (1.3)
GADA	37 (1.6)
IA-2A	10 (0.4)
ZnT8A	9 (0.4)
**CD Aab+**	**61 (2.7)**
IgA-tTG	53 (2.3)
IgG-tTG	52 (2.3)
**AITD Aab+**	**99 (4.4)**
TPOA	54 (2.4)
THGA	86 (3.8)
